# Electric Imaging through Evolution, a Modeling Study of Commonalities and Differences

**DOI:** 10.1371/journal.pcbi.1003722

**Published:** 2014-07-10

**Authors:** Federico Pedraja, Pedro Aguilera, Angel A. Caputi, Ruben Budelli

**Affiliations:** 1 Departamento de Biología Celular y Molecular, Facultad de Ciencias, Universidad de la República, Montevideo, Uruguay; 2 Departamento de Neurociencias Integrativas y Computacionales, Instituto de Investigaciones Biológicas Clemente Estable, Montevideo, Uruguay; University of Tübingen and Max Planck Institute for Biologial Cybernetics, Germany

## Abstract

Modeling the electric field and images in electric fish contributes to a better understanding of the pre-receptor conditioning of electric images. Although the boundary element method has been very successful for calculating images and fields, complex electric organ discharges pose a challenge for active electroreception modeling. We have previously developed a direct method for calculating electric images which takes into account the structure and physiology of the electric organ as well as the geometry and resistivity of fish tissues. The present article reports a general application of our simulator for studying electric images in electric fish with heterogeneous, extended electric organs. We studied three species of Gymnotiformes, including both wave-type (*Apteronotus albifrons*) and pulse-type *(Gymnotus obscurus* and *Gymnotus coropinae*) fish, with electric organs of different complexity. The results are compared with the African (*Gnathonemus petersii*) and American (*Gymnotus omarorum*) electric fish studied previously. We address the following issues: 1) how to calculate equivalent source distributions based on experimental measurements, 2) how the complexity of the electric organ discharge determines the features of the electric field and 3) how the basal field determines the characteristics of electric images. Our findings allow us to generalize the hypothesis (previously posed for *G. omarorum*) in which the perioral region and the rest of the body play different sensory roles. While the “electrosensory fovea” appears suitable for exploring objects in detail, the rest of the body is likened to a “peripheral retina” for detecting the presence and movement of surrounding objects. We discuss the commonalities and differences between species. Compared to African species, American electric fish show a weaker field. This feature, derived from the complexity of distributed electric organs, may endow Gymnotiformes with the ability to emit site-specific signals to be detected in the short range by a conspecific and the possibility to evolve predator avoidance strategies.

## Author Summary

Sensory imaging is a relevant issue in perception studies which is not yet fully understood. A specific sensory carrier's characteristics and how it interacts with pre-receptor structures to shape images are key aspects of all sensory systems. Comparative study leads to general concepts and a specialized jargon. Electric fish are widely used models for imaging studies and have led to important contributions in imaging research. We highlight the diversity of electric organ discharges as a source of different carriers subserving this active electric sense. Site specific differences in the organization of the electric organ of pulse Gymnotiformes results in a multi-directional “illumination” of objects in the surrounding environment. However, in both African and American species, there is a foveal region where the fields and the electric images show coherent waveforms that simplify the neural algorithms required for processing object images with high resolution. In addition, in American species the electric organs generate a complex field near the skin. This complex field tends to a dipolar form as it fades with distance from the electric organ, not very far away from the body. These features may have evolved as a cryptic adaptation of the electromotor system to deal with electroreceptive predators.

## Introduction

Weakly electric fish show two electrosensory modalities [1], [2] supported by the presence of two types of electroreceptors sensitive to transcutaneous electric fields [3]. Passive electroreception, shared with many aquatic animals, allows the perception of electric fields produced by external electric sources, for instance the muscles of prey or predators, or the electric signals of neighboring electric fish. Active electroreception [1], [2], [3] evolved independently in African and American electric fish and it is based on the selective tuning of electroreceptors to the waveform of the self-emitted electric field generated by the activation of an electric organ (EO). In active electroreception, objects with impedance different from water induce perturbations in the electric field generated by the self-generated electric organ discharge (EOD) [4]. Object-dependent variations of the self-generated field across the skin are considered “electric images”, conveying information that allows the detection, identification, discrimination and recognition of the elements present in the surrounding environment [5], [6], [7], [8], [9], [10].

Modeling the electric field and images generated by fish contributes to the better understanding of the pre-receptor conditioning of electric images, which in turn is the key to unravel peripheral encoding of electrosensory inputs. Two main strategies have been used to study electric imaging with complex EOs. On the one hand, Caputi and Budelli [6] developed a “direct”, bottom up model taking into account the structure and physiology of the EO and the geometry and conductivity of the fish body. On the other hand, Rasnow, Assad and MacIver [11], [12], [13], [14], and more recently Babineau and col. [15] used a more pragmatic strategy, finding the appropriate internal sources that matched the external field.

The first strategy has the advantage of having solid foundations in experimental measurements of the electrogenic sources and fish body impedance [16]. It has also the advantage of filling the gap between the knowledge of the electrogeneration mechanisms and the generation of electric images [6], [17], [18], [19], However, calculating the whole field of realistic 4-dimensional scenes (three dimensions of space plus time) with the “finite element” model would imply a very large computational demand. The second strategy, based on the boundary element method (BEM) has the advantage of providing faster and accurate calculation of electric images. Thus, our modeling is optimized by combining both strategies. Instead of calculating the whole field, we approximated the electric image applying the BEM in a new simulator that uses experimentally measured electromotive forces, internal conductivity and the geometry of the fish body (see the thesis by Rother [20] and ‘The Model’).

A first set of simulations was carried out on the electric sense of *Gnathonemus petersii*, an African Mormyrid fish. These fish have a localized EO situated close to the tail, that is activated synchronously, yielding a very brief EOD which facilitates the analysis of the results [8], [16], [21]. However the EO of American electric fish is distributed along the fish body, making the characterization and interpretation of images a much more complicated task [13], [14], [15], [22], [23].Thus, more recently we addressed the challenge of modeling the EO and the EOD of *Gymnotus omarorum*, a species where the electrogeneration mechanisms have been extensively studied [23]. These models, together with experimental results, have helped to understand the role of the fish's body on image formation as well as the peripheral encoding of object impedance [24], geometrical characteristics of the object [7], [24], [25], the object's distance and position [7], [8], [24], [26], [27].

Electroreceptor sensitivity and distribution are fundamental in the transformation of the electric image into a “neural image”. Strong evidence supports the existence of an “electrosensory fovea”. The presence of this region was first proposed based on evidence arising from the modeling of the electrogenic system of *G. omarorum* [6] and experimentally confirmed in *G. omarorum* [28], [29], [30], *G. petersii* [31], [32] and other species [33]. The electrosensory mosaic of the perioral region has the highest density and variety of receptors. This region has a large central representation and is stimulated by a relatively large, coherent and iso-oriented electrosensory carrier [29], [30], [34], a feature that has been described in those species by our previous modeling studies [16], [21], [23]. Nonetheless, it still remains unknown how the spatiotemporal complexity of the field and the electroreceptor type distribution (both characteristic of each species) contribute to the electrosensory encoding of the surrounding scenes. To unveil this issue it is necessary to understand both the common and the diverse mechanisms of electrosensory imaging across species.

In this paper, we explore these aspects through realistic modeling. The extension of our previous studies in *G. omarorum* [23] and *G petersii* [16], [21] to other Gymnotiformes species with different EOD complexity, has allowed us to show the capabilities of the simulator for calculating electric fields and images in all functional types of electric organs and therefore its potential as a tool for exploring active electrolocation and electrocommunication. The chosen species cover almost the whole spectrum of complexity of electric imaging strategies: a) pulse type EOD emitted by a localized EO (represented by *G. petersii*); b) a wave type EOD emitted by a distributed EO (represented by *Apteronotus albifrons*) and c) a wavelet type EOD represented by *G. omarorum* and two other species with different degrees of waveform complexity. *Gymnotus obscurus* shows an almost monophasic EOD, with a very simple spatial organization of the electric organ, while *Gymnotus coropinae* shows a multi-phasic and very complex spatiotemporal organization [35], [36], [37].

We applied the model to investigate: a) how the electromotor organization influences the range of electroreception and electrolocation in different species and b) the differences in electroreception mechanisms between rostral and other body regions. Our analysis suggests that Gymnotiformes may have a shorter range of electrolocation and electrocommunication than African mormyrid fish. Our study has confirmed the fovea - body differences of the field and images in the new species studied and explains how differences in EO structure and body geometry, together with a certain organization of the sensory mosaic, provide functional advantages for the corresponding electrosensory organization.

## Results

In this article, we compare the electric field generated by two pulse type and one wave type Gymnotiform fishes with different EOD complexity, with the previously studied *G. petersii* and *G. omarorum*. We addressed the following points: 1) how to calculate the equivalent source distribution based on experimental measurements, 2) how the complexity of the EOD determines features of the electric field surrounding the fish and 3) how the basal field determines the characteristics of electric images.

### From air gap recordings to source distribution

The coordinated activation of electrocytes or nerve fibers generates longitudinal currents that, flowing through the external media, generate the electric field due to the EOD. Although the EOD associated field may change with the sensory scene and particularly with water conductivity, we have shown that in most cases the EOD can be represented by an equivalent source which is characterized by the voltage generated in air and the impedance of contiguous parts of the fish's body [38]. This series of voltage values are a species specific invariant that can be used for calculating external fields [6]. For the localized EO of *G. petersii* we used a single dipole to simulate fields and images [16], [21]. However as Gymnotiformes have a distributed EO, discharging different waveform at different regions, a multi-poles approach is required. Then, we experimentally determined the voltages generated by contiguous parts/sections? of the fish body by measuring the difference of voltage between electrodes (air gap) while the fish were held in air [38]. These differences are generated across the body region encompassed between the electrodes.

**Figure 1:**
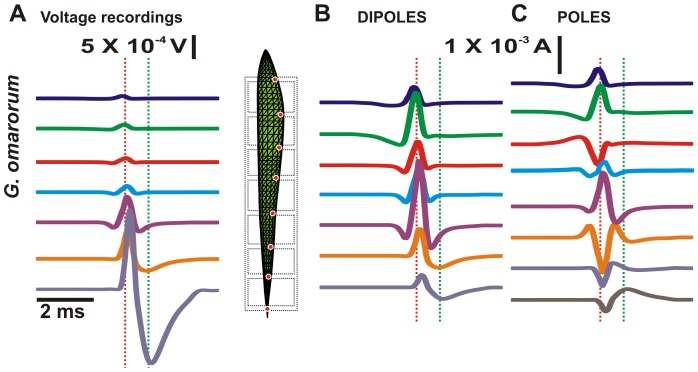
Voltages, dipoles and poles for *G. omarorum*. (A) Recorded potential differences through the air gaps. (B) Rostral poles of the dipoles calculated from the recorded potentials, fish resistivity and fish morphology. The diagram between A and B represents the fish in the multiple air gap. Red dots represent the position of the poles in the model. (C) Poles calculated from the dipoles as a function of time. The red and green dotted vertical lines represent the positive peak of the htEOD and the negative peak respectively.

The procedure is exemplified for *G. omarorum* in Figure 1, which shows in A the voltage recordings across the 7 air gaps (see data for the other species in Figure S1). Assuming that the voltage recorded from each air gap is produced by two poles of current sources of opposite polarity (dipoles) situated at either end of the body region (source and sink), the electric current can be calculated as V/R; where V is the recorded voltage and R is the longitudinal resistance of that part of the fish body (calculated according to tissue impedance and fish body geometry, see The Model). The time courses of longitudinal currents generated by the 7 rostral poles are presented in column B. Since the pieces of the fish body are contiguous and aligned longitudinally and are thus limited by a common plane, the currents supplied by the poles lying on the same plane can be reduced to a single entity by simple addition of their magnitudes, and the EO can be represented by a set of 8 poles (Figure 1C). Note that: a) the voltages increase rapidly towards the tail, b) waveforms are characteristic of each body part, and c) there is a delay between homologous peaks at different regions. This last occurs because the neural coordination mechanisms do not provide a perfect synchronism between EO regions [17], [39], [40], [41]. However, due to impedance matching, the maximal current contribution to the external field is provided by the central and caudal body regions (Figure 1B). In consequence, the poles invert their polarity at the limits of the central region of the fish, where they also show maximal absolute values (Figure 1C violet and orange traces).

Data was obtained for several species using this method, for which *G. obscurus* was the simplest case. For this fish, the voltage signal consists of a main positive component, increasing in amplitude and appearing with increasing delay as EO activation travels rostro-caudally. At the tail region there is a small negative component. Despite this apparent simplicity, the poles show complex waveforms illustrating the effect of the progressive shift of the positive peak onset from head to tail (Figure S1, [36]).

*G. coropinae* is the most complex case. This fish exhibits a large expansion of the EO at the head. Thus, besides the pattern already described for *G. omarorum*, *G. coropinae* shows a strong source that generates a different waveform starting significantly earlier than that generated by the rest of the EO (Figure S1, [37]).

*A. albifrons* is a wave type fish, with a neural EO. The magnitudes of the poles reach their maximum in the second and seventh defined body regions, due to the highly synchronous discharge of the EO. The most rostral and caudal dipoles are negligible. Currents from poles 3 to 6 are mild, but not negligible; if they were so, we would be able to simulate the field generated by the EOD by two distant poles (Figure S1 [42]).

Comparing the different pulse species we should stress that while heterogeneity plays a very important role at the transition between the head and central regions, the relationship between electrocyte number and internal resistance plays a major role at the transition between the central and tail regions [6]. In *A. albifrons*, the similarity and the almost synchronous discharge of different regions of the EO results in two major poles, at the head to central and central to tail transitions.

### From sources to electric fields

Using the BEM method (see The Model), we calculated the maps of electric potentials and fields, either in water or across the skin. The modeled field and images are multidimensional, including spatial and time dependent aspects. Thus we represent images as series of images profiles, defined as the transcutaneous voltages along a line on the skin, each element of the series corresponding to a given time. In certain cases images are represented as transcutaneous voltages, as a function of time at a given skin site. The drop of voltage and fields in water are shown in the same way.

**Figure 2:**
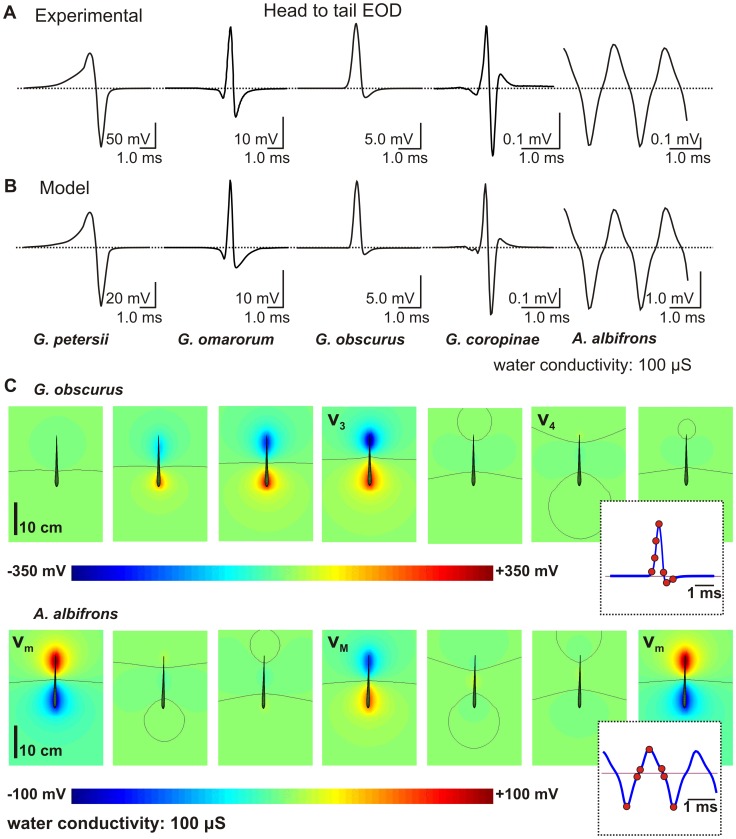
Head to tail EOD waveforms and electric potential in a horizontal plane. (A) The experimental htEOD recording across the species (B). The htEOD recording calculated using the BEM model. Dotted line indicates zero voltage. (C) *G. obscurus*: three instants before the positive peak, the positive peak, an instant between the positive peak and the negative peak, the negative peak and one instant later. *A. albifrons* at the peak of the negative wave of the htEOD, two instants close to the zero crossing between the negative and positive peaks, at the peak of the positive wave, two instants close to the zero crossing between the positive and negative peaks and again at the peak of the negative wave. Black lines indicate the points where the potential is zero. The insets show the htEOD waveform at the selected instants (red dots).

Our first aim was to check the accuracy of the model by reproducing the far field and the head to tail recordings as used in taxonomic and evolutionary studies [43]. In a previous paper, we checked that in *G. omarorum*, the simulated field fits the experimentally determined one [23]. Here we compare the simulated and experimental head to tail EOD (htEOD) for the studied species (Figure 2A and B). Since the experimental recordings were obtained by different authors in different tanks [35], [36], [44] and the simulations were calculated in an infinite medium, we focused only on the reproduction of the waveform, which, in fact, is outstandingly accurate. The main difference between *G. petersii* and American fish is that while in mormyrids wave transitions occur synchronously, in all Gymnotiformes the heterogeneity and asynchrony between the different regions of the EO generate differences between the near and the far field.

Furthermore, as explained in the appendix of a previous paper [23], in a three dimensional view, either the head or the tail can be enclosed by an ovoid shaped surface of zero potential, while another zero potential surface tending to infinite crosses the body at an intermediate level (Figure 2, C and Figure S2). This implies that different htEOD waveforms are recorded when the electrodes are either far or close to the body.

**Figure 3:**
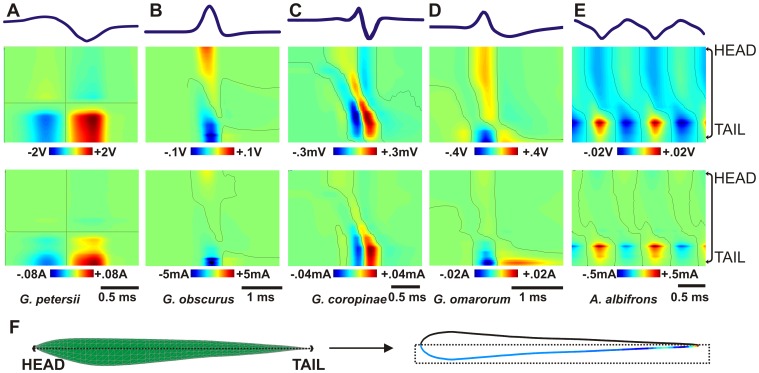
Electric potentials and fields perpendicular to the fish skin on a horizontal plane. (A) *G. petersii* (B) *G. obscurus* (C) *G. coropinae* (D) *G. omarorum* (E) *A. albifrons*. The top row shows the htEOD waveforms recorded in air as a reference. The second row shows the potential along a horizontal line on the skin as a colormap: x axis represents time along the EOD and y axis represents the position on the skin. Reversal points in black. The third row shows the transcutaneous currents using a similar representation. (F) schematic representation of the localization of the skin section in a lateral view (left) and seen from above (right). We have used the body profiles of *G. omarorum* but these are similar in the other fish.

A second piece of evidence confirming the accuracy of the model results from the comparison of the modeled sinks and sources on the fish skin with published experimental data [13], [43], [45]. We calculated the voltage as a function of time, measured along a lateral horizontal line on the fish skin (Figure 3 middle row). These maps indicate the presence of contiguous regions of different polarities, separated by zero lines (black lines). In *G. petersii* (Figure 3A), there is only one zero line that stays fixed in the same point. In Gymnotiformes the zero lines move from rostral to caudal regions as the EOD progresses, as expected by the progressive activation of the EO [39], [41]. The simplest case is that of *G. obscurus* (Figure 3B), having an almost monophasic time course. The most complex case is *G. coropinae* (Figure 3C), reflecting the presence of multiple generators with asynchronous evolution of the source (reddish) and sink (bluish) positions along the fish. *G. omarorum* (Figure 3D) and *A. albifrons* (Figure 3E) are intermediate cases. The bottom row of Figure 3 shows the species-specific transcutaneous current profiles, which are proportional to the strength of the field (the voltage gradient) perpendicular to the skin [24].

**Figure 4:**
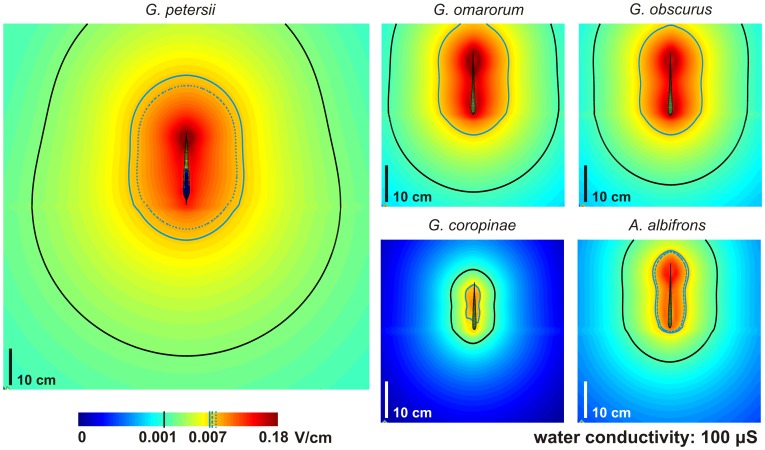
Comparison of maximum fields along the EOD. The color maps represent the maximum absolute value of the field at each point of space computed for the whole time course of the EOD. Purple lines show the experimentally obtained thresholds for active electrolocation for G. omarorum (continuous line); *G. petersii* (dotted line); and *A. albifrons* (dashed line). For the sake of comparison, in every fish we plot (continuous lines) the threshold values of active (in sky-blue) and passive (in black) electroreception, corresponding to those experimentally determined for *G. omarorum* (values taken from [46], [47], [48], [49], [51]).

Finally we compared the strength of the fields close and far from the fish. Figure 4 shows the maps of maximum field at each point, on a logarithmic scale. We marked (in purple) the experimentally obtained thresholds for active electrolocation for *G. omarorum* (continuous line), *G. petersii* (dotted line); and *A. albifrons* (dashed line). For the sake of comparison, we also plot the threshold values of active (in sky-blue) and passive (in black) electroreception of *G. omarorum*. We found that the strength of polarization of a neighboring object differs among species, the range for *G. petersii* being much larger than the range for the Gymnotiformes species, and that of *G. coropinae* being the smallest of all. This may explain the differences in electrolocation ranges found in the literature [46], [47], [48], [49]. In addition the data also suggest that the distance for detecting a field produced by a conspecific with an EOD of the same amplitude would be significantly smaller in Gymnotiformes, reaching the lowest level in the species with the most complex EO (*G. coropinae*).

### From fields to images: Imaging mechanisms studied with small metal spheres

The clue to understand electric imaging is to realize that object polarization is the source of electrosensory signals, resulting from the change in the electric field determined by the presence of an object. This change (perturbing field) is defined as the field resulting from subtracting the electric field in the absence of the object (basal field) from the electric field in its presence (stimulating field) [2].

**Figure 5:**
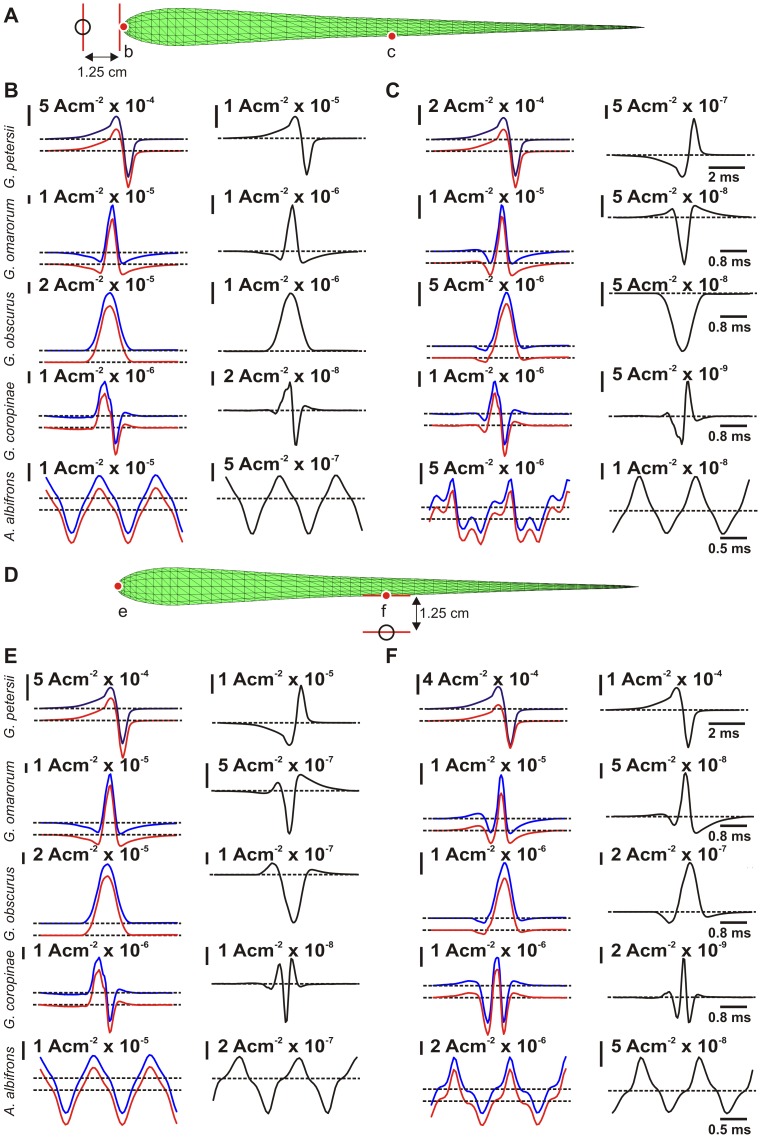
Time course of the image when the object is placed before the fovea and at the side of the body. (A) Diagram of the scene. The red dots marked as b and c correspond to the places where the traces shown in B and C were calculated. (B) Time courses at the fovea. Left column: Time courses of transcutaneous currents in the absence (red), and in the presence (blue) of an object facing the fovea. Right column: The image calculated as the difference between the traces on the left (black). (C) Time courses for transcutaneous currents with and without an object situated laterally. (D) Diagram of the scene. The red dots marked as e and f correspond to the places where the traces shown in E and F were calculated. (E) Time courses at the fovea. Left column: Time courses of transcutaneous currents in the absence (red), and in the presence (blue) of an object facing the side. Right column: The image calculated as the difference between the traces on the left (black). (F) Time courses on the side, color-coded as above.

Depending on the object location and the fish species, the time courses of the object perturbing and stimulating fields may, or may not, be equal. Figure 5 compares the basal and stimulating field and their difference (object perturbing field) at the head and the side of the fish, when an object is placed in front of one of the recording positions. In all cases the time courses of the perturbing field in front of the object are equal but have opposite polarity with respect to the other recording point. This difference in polarity of the object perturbing field is due to the ‘Mexican hat’ center-surround image profile ([7]): when the image of an object formed at the skin has a given polarity representing the center of the object, the surrounding skin will see an image tending towards the opposite polarity, corresponding to the ‘brim’ or the trough of the Mexican hat profile.

In mormyrids, the time courses of stimulus and perturbing fields are equal, but in Gymnotiformes they are often different. This implies that for a pure resistive object, the object position may be encoded not only by the spatial pattern but also by the waveform pattern at different spatial locations. It is clear from the Figure 5 that in all Gymnotiformes there is little difference between perturbing and basal field at the head, when an object is facing the recording point (Figure 5B). In contrast, for the same scene, there are important differences when the recording site is on the side of the fish (Figure 5C). When the object faces the side of the fish, perturbing and basal fields are different at both recording sites (Figure 5E and F). To study this phenomenon in detail, we compared the images generated by objects near the head or on the side of the fish.

#### Spheres facing the rostral zone

**Figure 6:**
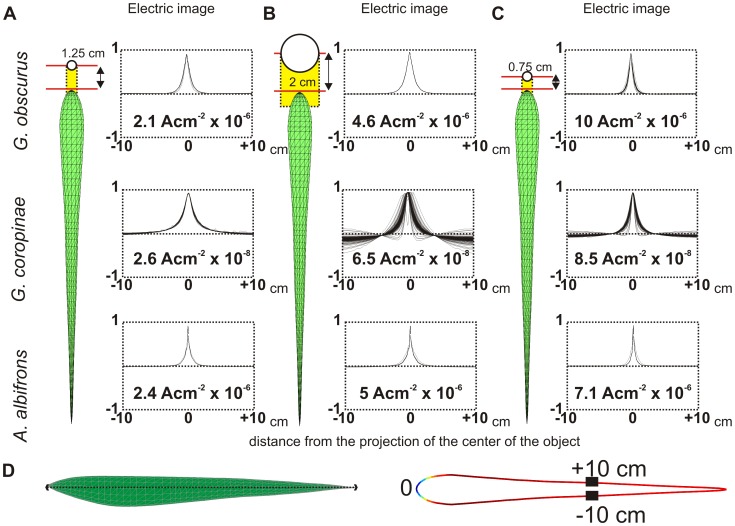
Image profiles for spheres of different size facing the fovea. Amplitude image profiles for *G. obscurus*, *G. coropinae* and *A. albifrons* when (A) a small (0.25 cm radius) and (B) a large sphere (1 cm radius) face the fovea at when the distance between the skin and the surface of the sphere is 1 cm) and (C) when the small sphere faces the fovea at a shorter distance (0.5 cm). The plot shows the profile for the entire EOD normalized by the absolute maximum of each peak. The yellow area indicates the projection of the object on the skin. Note the different shapes for *G. coropinae*. (D) Schematic representation of the localization of the skin section in a lateral view (left) and seen from above (right), for *G. obscurus*. See Figure S3 for the complete image.

Figure 6A (and Figure S3) shows, for the same scene as in Figure 5A, a series of electric images profiles. In all fishes, the image has a symmetrical center-surround opposed profile centered in front of the object. When a sphere of 0.25 cm radius is placed at 1 cm from the skin, the image profile is almost constant, changing only in amplitude. Thus, there is an almost perfect superposition of the normalized image profiles (Figure 6A and S3). Note that: a) the amplitude of the image in Gymnotiformes is at least one order of magnitude smaller than in *G. petersii* and b) in *G. coropinae* the images profiles are 2 orders of magnitude smaller than in the other studied species (Figure 5, 6 and S3). These features of the image contrast with those observed for a larger object (1 cm radius) or for a similar object located closer to the fish (0.5 cm from the skin). For the larger sphere, the widths of the image profiles are similar to those of the small sphere at the same distance. The superposition shows very similar image profiles except for *G. coropinae*, in which the discrepancy indicates that the spatial profiles are changing along the EOD (Figure 6B and 3S). For the small sphere located closer, the width of the profile decreases and the superposition is a bit less perfect in all fish, but this is most marked in *G. coropinae* (Figure 6C and S3).

#### Spheres facing the lateral zone

**Figure 7:**
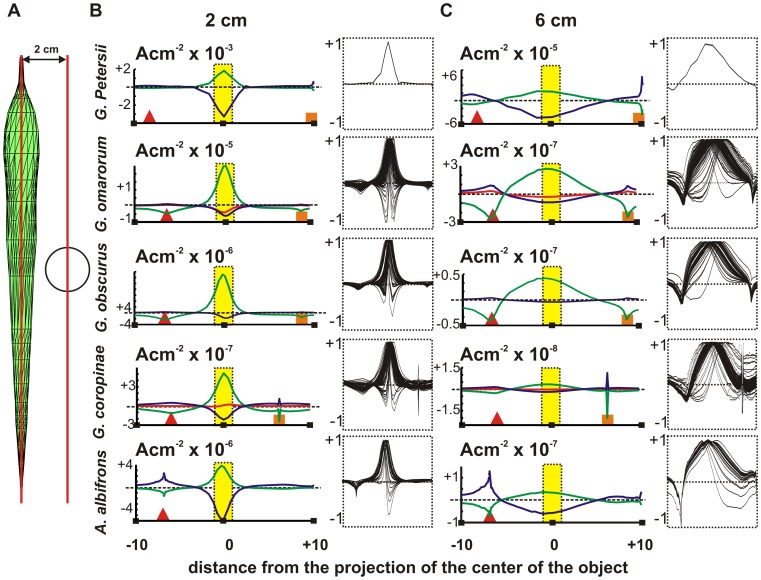
Images of a sphere facing the middle portion of the fish body. (A) The diagram shows the relative position of the sphere when the distance to the longitudinal axis is 2 cm. (B) Each row shows the image profiles of a sphere situated at 2 cm from the sagittal plane for the studied species. (C) Image profile with the sphere at 6 cm. The plots show the profiles at the peaks of the htEOD waves: positive peak (green) and negative peak (blue). Also shown are the rostral positive peak (red) for *G. coropinae* and the first negative peak (red) for *G. omarorum*,. Insets show the superposition of normalized profiles (divided by the maximum absolute value along the EOD). The triangles and squares indicate the fovea and the tail tip respectively; the yellow area indicates the object projection on the skin.

Figure 7A compares the image profiles of a 1 cm radius metal sphere, placed 2 cm away from the fish midline, for the main peaks of the htEOD in the five studied species. While in *G. petersii* and *A. albifrons* the profiles of the main peaks are coincident, in pulse Gymnotiformes there is a spatial shift. Furthermore in all Gymnotiformes, the transitions between the main peaks are characterized by clearly different biphasic profiles, as illustrated in the insets. This indicates changes in the direction of the transcutaneous field. When the object is moved away from the fish body, the image profiles increase in width and decrease in amplitude and the changes along the EOD are attenuated (Figure 7B). Differences between profiles increase from the side of the head up to the 3/4 of the fish length measured from the snout (Figure S4, S5, S6, S7, S8).

## Discussion

Electric fish use the EOD as a signal carrier for object exploration and communication. The electromotor pattern varies across species, implying different strategies of image generation, which in turn may imply differences in the organization of the sensory pathway. In this article we combine modeling and comparative analysis to explore how the commonalities and species-specific differences in electric imaging may depend on the different organization of the electromotor systems. The main differences between African and American electric fish are caused by the extension and complexity of the EO. The increased complexity of the EO of American electric fish leads to the hypothesis that they may detect object location through waveform analysis. The simple organization of African mormyrids results in a long communication range while complexities may provide American fish with the possibility to emit site-specific signals to be detected in the short range as well as the possibility to evolve cryptic predator avoidance strategies.

### The role of electromotor strategy on sensory imaging

Our model results fit well with experimental recordings of near and far fields for different species, confirming its validity. First we reproduced the time course of the htEOD. One of the limitations of the model is that the simulations cannot account in a precise way for the tank borders, and for this reason all calculations were performed as if the fish were in an infinite homogenous media. This may account for the differences in the amplitude of the htEOD and the differences in the time courses of the early components of *G. coropinae*.

In active electrolocation, the stimulus to the sensory mosaic in the presence of an object is the stimulating field: the sum of the basal field plus the object perturbing field [10], [50]. Here we have shown that the time course of the field polarizing the object is species specific, as well as dependent on the object location with respect to the fish's body.

The simplest case is represented by pulse mormyrids. These fish have a localized EO at the caudal peduncle that is synchronously activated yielding a very brief EOD. Here we confirmed previous experimental and modeled data indicating that the basal field, stimulating the mosaic and the object perturbing field have the same time course when the scene is formed objects that are resistive only [7], [9], [16], [20], [21], [24], [45].

In contrast, Gymnotiformes show a distributed EO in which the time course of the regional electromotor sources is either shifted in time (as it is the case of *A. albifrons* and *G. obscurus*) or shows a characteristic regional waveform (as it is the case of *G. omarorum* and *G. coropinae*). Under the assumption of linearity we have extended the initial model based on a localized EO [20] by calculating object polarization and basal fields as the sum of the effects of eight equivalent poles that change in magnitude with time. In order to calculate poles we started from experimentally recorded voltages with the fish in air and the geometric profile of the fish body [6]. Since voltage amplitude, time course and body shape are species specific, the resultant polarizing field from each pole is also species-specific. Moreover, since the relative distance from the object site to each pole varies with the position of the object, object polarization is also site-specific for Gymnotiformes fish.

### Commonalities and differences in active imaging

The first commonality is that all studied species show a short electrolocation range. However, there are differences between fish generating large (*G. petersii*) and small (Gymnotiformes) electromotive forces and having synchronous or heterogeneous discharges. We found a good agreement between the electrolocation ranges predicted by the extrapolation of the detection threshold of *G. omarorum* [51] to the electric field of *A. albifrons* [46] and *G. petersii* [47], [48], [52]. It is important to note that as the polarization distance increases, the projection distance also increases which in turn reduces the image amplitude. Therefore, the exclusive consideration of object polarization leads to an over-estimation of the electrolocation range. Similarly, we have found that the extrapolation of the communication range of *G. omarorum* is in the experimentally determined range for *G. petersii* [53], [54]. Finally, the EO complexity increases the probability of the cancelation of the far field, produced by neighboring regions of the fish, as occurs in *G. coropinae*.

A second commonality is the peculiar characteristic of the imaging mechanism at the perioral region. This finding is consistent with the general hypothesis that the perioral region and the rest of the body play different sensory roles [55]. While the “electrosensory fovea” might be used for resolving details of the explored objects, the rest of the body might be used as a “peripheral retina” for detecting the presence and movement of objects. These results generalize some experimental observations about object imaging at the fovea of *G. omarorum* [55], [56]. At the fovea the details of the size and shape of the object are only represented in the image profiles when the object is very close. In fact, as shown in Figure 6, a small and a large sphere placed 1 cm away from the skin show the same image shape, differing only in amplitude, in all studied fish with the exception of wave transitions in *G. coropinae*. This difference in image formation is due to the curved shape of the snout and the mandibular region [45], [55], [56]. In this region the object's presence generates a similar image profile in each instant of the EOD. When an object is large enough for its edges to exceed this region (the larger or the closer sphere, in Figure 6B and C) the image profiles change in shape. *G. coropinae* has a slender body; hence this region is narrower in this species.

A third commonality is that the constant temporal profile of images observed for most species near the fovea facilitates capacitance discrimination as previously shown in *G. petersii* [57], [58] and *G. omarorum* [59]. The constancy of the time course of the EOD at the perioral region facilitates the identification of the changes in the stimuli induced by capacitance since within a fringe surrounding the fovea the only cause for a discrepancy between the time courses of the object polarization and the basal field is the complex nature of the object impedance. To discriminate qualia (as color, in vision), it is necessary to have at least two types of receptors, responding differently to the stimulus that reaches the sensory surface. In vision the light is composed by photons of different frequencies that differentially stimulate the three types of cones. Each of these images is characterized by a particular spatial pattern of amplitude or shape. In electroreception, qualia relates to the differential responsiveness of electroreceptors to the time course of the local EOD. Tuberous electroreceptors can be classified in various subtypes depending on the species. *G. petersii* has two subtypes [60], [61], in the genus *Gymnotus* there are four [62], [63], [64]; and in other Gymnotiformes there are at least two [65], [66]. This has led to the idea of “electric color” in pulse fish [24], [57], [67]. This shared characteristic of several subtypes of electroreceptors across species may suggest the use of similar algorithms for decoding object impedance. More intriguing is the possibility of “electric color” decoding by wave fish. In this case, the brain should compute the image as spatial changes in amplitude and phase of a sine-wave like stimulus [66].

Similarly to other sensory modalities, the trunk of the fish body may act as a peripheral electrosensory “retina” where most of the information coded deals with the presence, location, or movement of objects. Consistently with a smaller spatial resolution, images are also less sharp and may have more than one peak. For example, the presence of an object close to the fish's side can be detected both on the side and on the perioral region (Figures S10–S13 rows 2 and 3). This foveal stimulation by objects placed on the side of the fish was experimentally shown in *G. petersi* [68] and *G omarorum* [51]. Finally, for objects located away from the fish, image intensity on the side is relatively large, opening the possibility to decode the rough position of the object by waveform analysis, followed by an object tracking or avoidance responses to further inspect or to escape from the object [11].

### Advantages and disadvantages of having a complex EO

Self-generated fields should have enough magnitude to ensure electroreceptor stimulation by the transcutaneous current of the emitter and, in the case of electrocommunication, by conspecific fish. From an evolutionary point of view two different strategies can be distinguished. African fish increased the EOD power by packing a large number of flat electrocytes into their highly localized EO. These electrocytes are oriented in parallel with the large surface perpendicular to the main axis of the body and are almost synchronously activated by the central nervous system. In addition, the large cross-section of the high conductance body spreads the generated current rostrally, increasing the equivalent dipole measured at a distant position [21]. This may facilitate long range communication between conspecifics. The time course of the field, constant everywhere, assures that the variations of the local stimuli generated by a capacitive object, fall within a family of waveforms that depend only on the impedance of the object, suggesting the perception of “electric color” [24], [57].

American fish evolved EOs composed by numerous, large, and relatively separated electrocytes extended all along the fish's body. The simplest cases considered are *G. obscurus* and *A. albifrons*. In these species the regional EODs have almost the same temporal course all along the EO. At the fovea which is away from the EO, imaging mechanisms are overall similar to *G. petersii*. However, on the fish's side the time delay between the activation of the different regions leads to the described differences between the images of the same object when it is placed at different sites. *G. omarorum* and other pulse Gymnotiformes show regional EODs with different time courses. In addition to the rostro-caudal time shift described for *G. obscurus* and *A. albifrons*, most species of the genus *Gymnotus* and *Rhamphychthys* [69] show temporal-overlapping of neighboring sources whose time course is opposite. Among the studied species, *G. coropinae* shows the largest complexity. In this species the duration of the different components is shorter in relation to the delay between regions, therefore facilitating overlapping between generators of opposite sign [36], [70]. Furthermore, as a consequence of EO complexity, synchronous but opposed field generators in different regions of the fish cancel out relatively close to the fish's body. Since these two features appear to be disadvantages for electrolocation and electro-communication, what could be the advantage of this evolutionary strategy?

One advantageous functional consequence of EO complexity is the potential generation of site-specific signals in the near field, while maintaining a single species-specific EOD time course in the far field. This allows the fish to identify an object's position by analyzing waveform. In addition, the near field may be indicative of species gender. In the biphasic htEOD fish, *Brachyhypopomus gauderio*, three phasic site-specific near field EOD time courses may be used as communication signals during typical courtship displays [71], [72] [Silva and Caputi, unpublished].

Finally, a smaller far field with high frequency components may be used as an encrypting strategy to avoid predators [73]. *G. coropinae* and other members of the clade 1 [36], [44], [70] are small sized fish exhibiting multiple asynchronous sources, yielding a very complex near field and a much less extended far field (Figure 4). This feature allows *G. coropinae* to use its complex discharge mainly for active electrolocation while the same complexity reduces the far field potentials, allowing this fish to cryptically hide from predators. As a complementary feature, in order to secure active electrolocation, this and other members of this clade (e.g. *G. javari*) might also have evolved strong sources in the head with a rostral common pole at the midline and two lateral poles [36]. This rostral extension of the EO allows the fish to generate strong currents through the fovea but a small far field [36], [37], [43]. This design reduces the region where the electrosensory mosaic is best suited for waveform analysis.

## Methods

### Detailed spatial analysis of the EOD

The multiple air gap procedure was used to characterize the detailed spatio-temporal patterns of the voltage distribution along the fish, while allowing us to compare EOD waveforms generated by different regions of the fish's body [74]. Fish were suspended in air using a custom-made apparatus that holds the fish on a grill-like array of parallel wires. The wires in contact with the skin were perpendicular to the main axis of the body, one at each end of the fish and others at the limits of each of the explored regions. Voltages were simultaneously recorded between pairs of wires, amplified to reach adequate amplitude for similar quantization (bit resolution always larger than 8 bits), and sampled at 25 kHz. For the purposes of this investigation, seven regions of the fish's body were considered; their lengths were adapted to cover the whole length of the fish. Because of the different origins of the wave components, several recordings were obtained for each fish with different configurations before finding the montage yielding the most representative picture of the EOD pattern. In general, regions showing larger changes in voltages for any of the components were explored with a greater resolution. The length of the regions varied in integer multiples of 1 cm. In the multiple air gap condition, since load is absent, voltage recordings are considered good estimators of the equivalent electromotive forces generated by the different portions of the fish's body for the components that are directly activated by synaptic action.

### Modeling the electric organ

We used the air gap data obtained from *G. omarorum, G. coropinae, G. obscurus* (already published by Rodriguez-Cattáneo and cols., [36]) and data from *A. albifrons* obtained in the same way. To evaluate resistance of the internal tissues and the skin, we used previously available data for *G. omarorum* [6] and assumed the same specific resistivity in *G. obscurus*, *G. coropinae* and *A. albifrons*.

The voltage difference, between 8 consecutive transverse planes of the fish placed at different sites of the body are mainly produced by the regions of EO encompassed by these planes and, according to Ohm's law, it is equal to the current (I) flowing through the internal tissues between each pair of planes times the resistance (R) of that section of the fish's body. Then, from the resistance of the given section of the fish body and the measured voltage (V) across it, we were able to calculate the current causing the voltage drop.
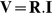
(1)This is based in the simplifying assumption that for a small longitudinal region of the EO the electrocyte population is homogeneous. Also according to the simplest assumption, electrocytes within a short segment of the electric organ are oriented similarly and fire almost synchronously. Thus in the model, the current generated by the series of identical dipoles -mimicking the electrocytes inside a cylindrical body slice- is equivalent to a dipole. This is because the rostral pole of one dipole adds with the caudal pole of the next caudal dipole: consequently, all the intermediate poles are canceled and the line of dipoles is equivalent to a single dipole with poles situated at the transverse planes limiting that piece of fish.

The longitudinal resistance (R) of a section of fish can be calculated from the geometry and resistivity (**ρ**)
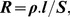
(2)where l and S are respectively the distance between recording electrode planes and the average cross-sectional area of the encompassed body portion

Hence:
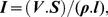
(3)The poles lying on the plane separating contiguous longitudinal pieces of the fish can be reduced to one by addition, and the EO can be represented by a set of poles equal in number to the planes limiting the experimentally studied regions of the fish. This method requires to identify whether there are abrupt transitions in the regional EOD waveform, and to place gap limiting planes at the transition points.

In the case of *G. omarorum*, *G. obscurus* and *A. Albifrons*, the anatomical and electrophysiological analysis indicates that the electrocytes are longitudinally oriented and thus, this analysis suffices for the representation of the EO. In the case of *G coropinae* [37] the variation of the electrocytes dipoles is not smooth enough and the EO is not oriented along a line. The head portion of the EO is the junction of four linear columns (two lateral and two central) arranged in an arrowhead shape [37]. To model EO complexity in this species, we considered 3 dipoles with a rostral common pole. The other three poles were located behind the opercula and at the abdominal wall. This approach produced similar results to the simulation obtained using a single dipole in the sagittal plane (Figure S9).

### Modeling electric images

Modeling of electric images was done using software developed by Diego Rother, advised by R. Budelli [20]. This model has two parts, a geometric reconstruction of the fish's body and a calculation of the transcutaneous field. Some later improvements to the Matlab codes used for geometric reconstruction of the fish's body and data presentation were introduced by Heric Rodriguez (unpublished results) and Juan-Ignacio Sanguinetti-Schek et al. [23].

The model was constructed under the following assumptions: 1) All media are ohmic conductors. This means that the vector representing the current density at the point *x* (***J*** (***x***)) is proportional to the vector electric field at the same point (***E***(***x***)).
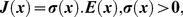
(4)The proportionality constant ***σ*** (***x***), referred to as “the volumetric conductivity at the point *x*” is always a positive scalar.

2) There are no capacitive elements. Therefore there is no charge accumulation at any point in space. In mathematical terms the variation of the density of charge (*ρ(x)*) is nil.
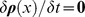
(5)


3) Given that the dielectric relaxation of the media is in general smaller than the minimum significant period of the EOD Fourier components, the model is an electrostatic approximation [75].

4) The fish and other objects are immersed in an infinite water medium. The shape of the fish body and objects are approximated by an external surface composed by triangles, allowing an approximation of the object shape that is limited only by the computation power available. Every object should be covered by a thin resistive layer (the skin in the case of the fish), which can be homogeneous or heterogeneous in resistance (magnitudes specified as desired).

The model is based directly on the charge density equation which, under our assumptions, implies that the charge generated by the sources (*f(x)*) is equal to the charge diffusion (*∇* ***J(x)***):
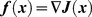
(6)and therefore
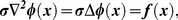
(7)where ***φ(x)*** is the local potential at the point *x*.

This differential equation, the so-called Poisson equation, can be solved for the fish boundary using the Boundary Element Method applied for wave fish by Assad [76]. Briefly, this method determines the boundary electrical distributions solving a linear system of 2N equations for N nodes, where, the unknown variables are the trans-epithelial current densities and potentials that correspond to each node (for a detailed description see [77]). The known variables were the location of the nodes, the location and magnitude of the poles representing the EO inside the fish, the conductance of the internal tissues, the skin and the water. It is important to note that, differently from Assad's method, a set of important constraints of the model were those posed by the electric organ equivalent sources that were experimentally measured using the air gap method. In the first instance, only trans-epithelial current densities and potentials are calculated at the “skin” nodes and these are then linearly interpolated in the triangles defined by the nodes. This allows the calculation of the potentials in the surrounding space.

## Supporting Information

Figure S1 **Voltages, dipoles and poles for:** ***G. Obscurus*** **(top)** ***G coropinae*** **(middle) and** ***A albifrons*** **(bottom).** (A) Recorded potential differences through the air gaps. (B) Rostral poles of the dipoles calculated from the recorded potentials, fish resistivities and fish morphology. The diagram between A and B represents the fish in the multiple air gap. Red dots represent the position of the poles in the model. (C) Poles calculated from the dipoles as a function of time. Red vertical line represents the positive peak of the htEOD, the green line indicates the negative peak and the blue line in *G. coropinae* shows the rostral positive peak.
(TIF)

Figure S2 **Electric potentials generated by the EODs in a horizontal plane.** (A) *G. omarorum*: The sequence shows from left to right: the first negative peak, two instants close to the zero crossing of the htEOD between the first negative and the positive peak, the positive peak, two instants close to the zero crossing of the htEOD between the positive peak and the last negative peak, and the negative peak. (B) *G. coropinae*: three instants before the rostral positive peak, the rostral positive peak, the caudal positive peak, two instants close to the zero crossing between the positive peak and the negative peak, and the negative peak. Black lines indicate the points where the potential is zero. The insets show the htEOD waveform with the selected instants (red dots).
(TIF)

Figure S3 **Image profiles for spheres of different size facing the fovea.** Amplitude image profiles for all the species when either a small (A) or a large (B) sphere faces the fovea at the same distance and when the small sphere faces the fovea at a shorter distance (C). The plot shows the profile for the main components of the EOD. The yellow area indicates the projection of the object on the skin. The inset shows the profile for the entire EOD, normalized by the absolute maximum of each peak. Color coding of the traces: *G. petersii*: negative peak (green), positive peak (blue); G omarorum: first negative peak (red), positive peak, (green), last negative peak (blue*); G. coropinae*: first negative peak (red), positive peak, (green), last negative peak (blue), *G. obscurus*: positive peak (green), negative peak (blue); *A. albifrons*: positive peak (green), negative peak (blue).
(TIF)

Figure S4 **Images of a sphere at 5 points on the side of *G. petersii.*** (A) The diagram shows the relative position of the sphere for each row, when the distance to the longitudinal axis is 2 cm. Each row shows the image profiles for spheres at 2 and 6 cm from the sagittal plane and at different positions along the longitudinal axis shown in the diagram. The plots show the profiles at the peaks of the htEOD waves : positive peak (green) and negative peak (blue). Insets show the superposition of normalized profiles (divided by its maximum absolute value along the EOD). The triangles and squares indicate the fovea and the tail tip respectively in each plot and the yellow area indicates the object projection on the skin. (B) Schematic representation of the localization of the skin section in a lateral view and seen from above, for a sphere placed close to the rostral region.
(TIF)

Figure S5 **Images of a sphere at 5 points along the side of** ***G. omarorum*** **.** Scheme and profiles as in Figure S4. The plots show the profiles at the peaks of the htEOD waves: negative peak (red), positive peak (green) and last negative peak (blue). Insets show the superposition of normalized profiles along the EOD. Note that the shapes of the images at the peaks of the waves differ mainly in the 4th line.
(TIF)

Figure S6 **Images of a sphere at 5 points along the side of** ***G. obscurus*** **.** Scheme and profiles as in Figure S4. The plots show the profiles at the peaks of the htEOD waves, positive peak (green) and negative peak (blue). Insets show the superposition of normalized profiles along the EOD. Note that the shapes of the images at the peaks of the waves differ mainly in the 3rd line.
(TIF)

Figure S7 **Images of a sphere at 5 points along the side of** ***G. coropinae*** **.** Scheme and profiles as in Figure S4.The plots show the profiles at the peaks of the htEOD waves: first negative peak (red), positive peak (green) and last negative peak (blue). Insets show the superposition of normalized profiles along the EOD. Note that the shapes of the images at the peaks of the waves differ mainly in the 4th line.
(TIF)

Figure S8 **Images of a sphere at 5 points along the side of** ***A. albifrons*** **.** Scheme and profiles as in Figure S4.The plots show the profiles at the peaks of the htEOD waves: positive peak (green) and negative peak (blue). Insets show the superposition of normalized profiles along the EOD. Note that the shapes of the images at the peaks of the waves differ mainly in the 4th line.
(TIF)

Figure S9 **Comparison of different models for EOs in** ***G. coropinae*** **.** Color maps show external potentials calculated using a single dipole (A), two symmetrical dipoles (B) and three symmetrical dipoles (C).
(TIF)
