# Supplementary figures and images for: Electric Imaging through Evolution, a Modeling Study of Commonalities and Differences
Source: PLoS Comput Biol. 2014 Jul 10;10(7):e1003722. doi: 10.1371/journal.pcbi.1003722 (PMC4091691; doi:10.1371/journal.pcbi.1003722)

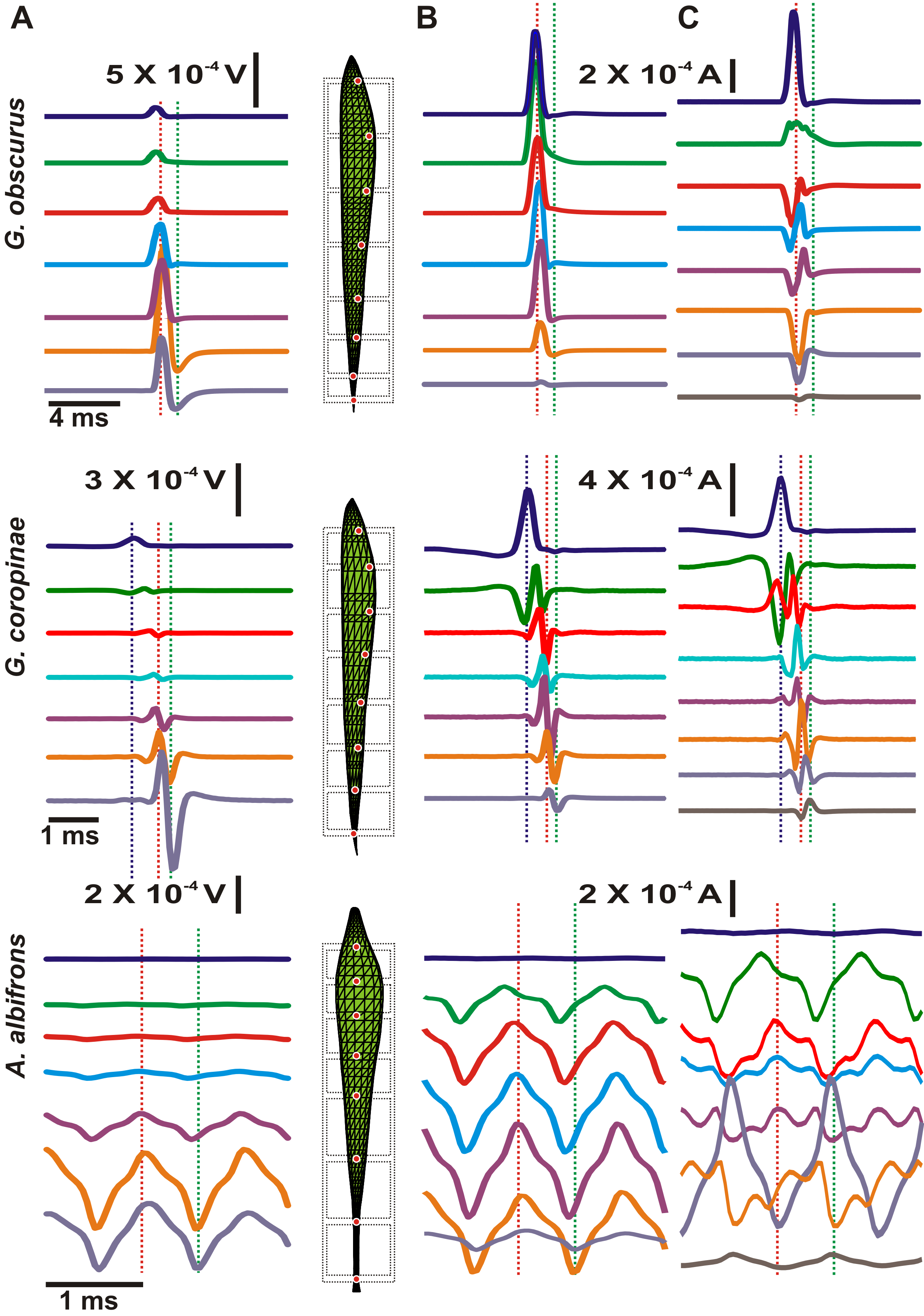

Supplement: Figure S1 — Voltages, dipoles and poles for: G. Obscurus (top) G coropinae (middle) and A albifrons (bottom). (A) Recorded potential differences through the air gaps. (B) Rostral poles of the dipoles calculated from the recorded potentials, fish resistivities and fish morphology. The diagram between A and B represents the fish in the multiple air gap. Red dots represent the position of the poles in the model. (C) Poles calculated from the dipoles as a function of time. Red vertical line represents the positive peak of the htEOD, the green line indicates the negative peak and the blue line in G. coropinae shows the rostral positive peak. (TIF) [file pcbi.1003722.s001.tif]

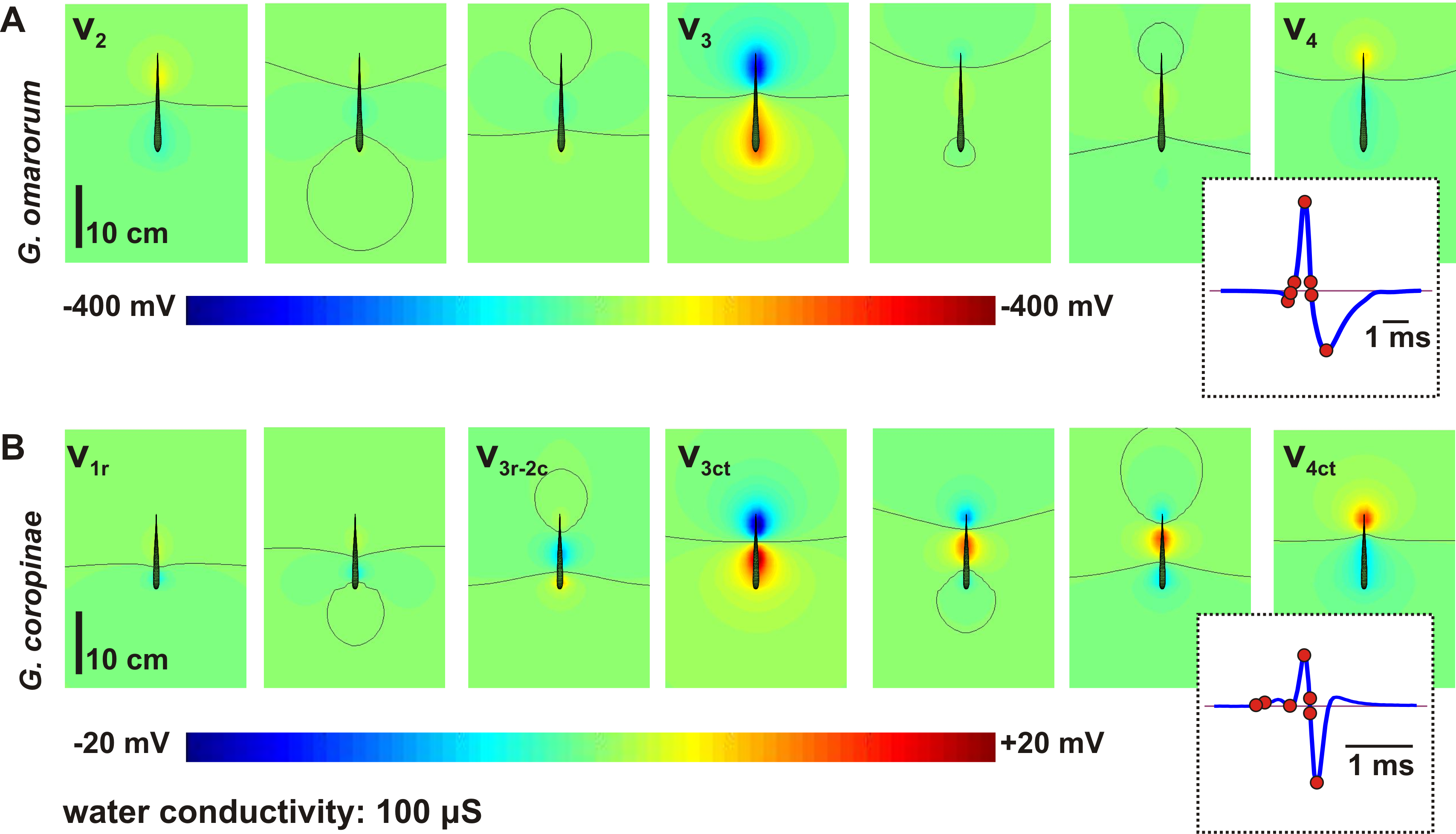

Supplement: Figure S2 — Electric potentials generated by the EODs in a horizontal plane. (A) G. omarorum: The sequence shows from left to right: the first negative peak, two instants close to the zero crossing of the htEOD between the first negative and the positive peak, the positive peak, two instants close to the zero crossing of the htEOD between the positive peak and the last negative peak, and the negative peak. (B) G. coropinae: three instants before the rostral positive peak, the rostral positive peak, the caudal positive peak, two instants close to the zero crossing between the positive peak and the negative peak, and the negative peak. Black lines indicate the points where the potential is zero. The insets show the htEOD waveform with the selected instants (red dots). (TIF) [file pcbi.1003722.s002.tif]

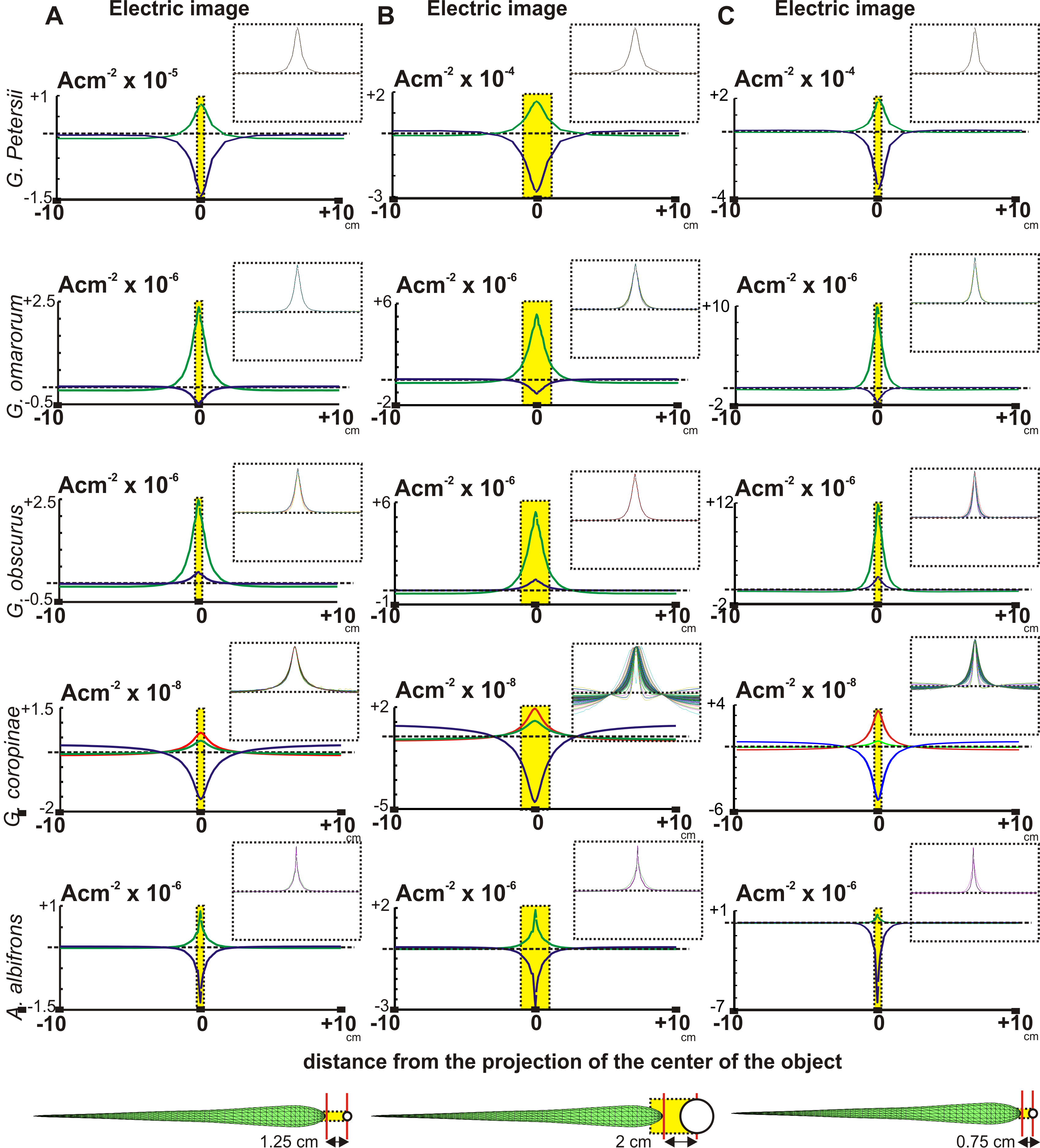

Supplement: Figure S3 — Image profiles for spheres of different size facing the fovea. Amplitude image profiles for all the species when either a small (A) or a large (B) sphere faces the fovea at the same distance and when the small sphere faces the fovea at a shorter distance (C). The plot shows the profile for the main components of the EOD. The yellow area indicates the projection of the object on the skin. The inset shows the profile for the entire EOD, normalized by the absolute maximum of each peak. Color coding of the traces: G. petersii: negative peak (green), positive peak (blue); G omarorum: first negative peak (red), positive peak, (green), last negative peak (blue); G. coropinae: first negative peak (red), positive peak, (green), last negative peak (blue), G. obscurus: positive peak (green), negative peak (blue); A. albifrons: positive peak (green), negative peak (blue). (TIF) [file pcbi.1003722.s003.tif]

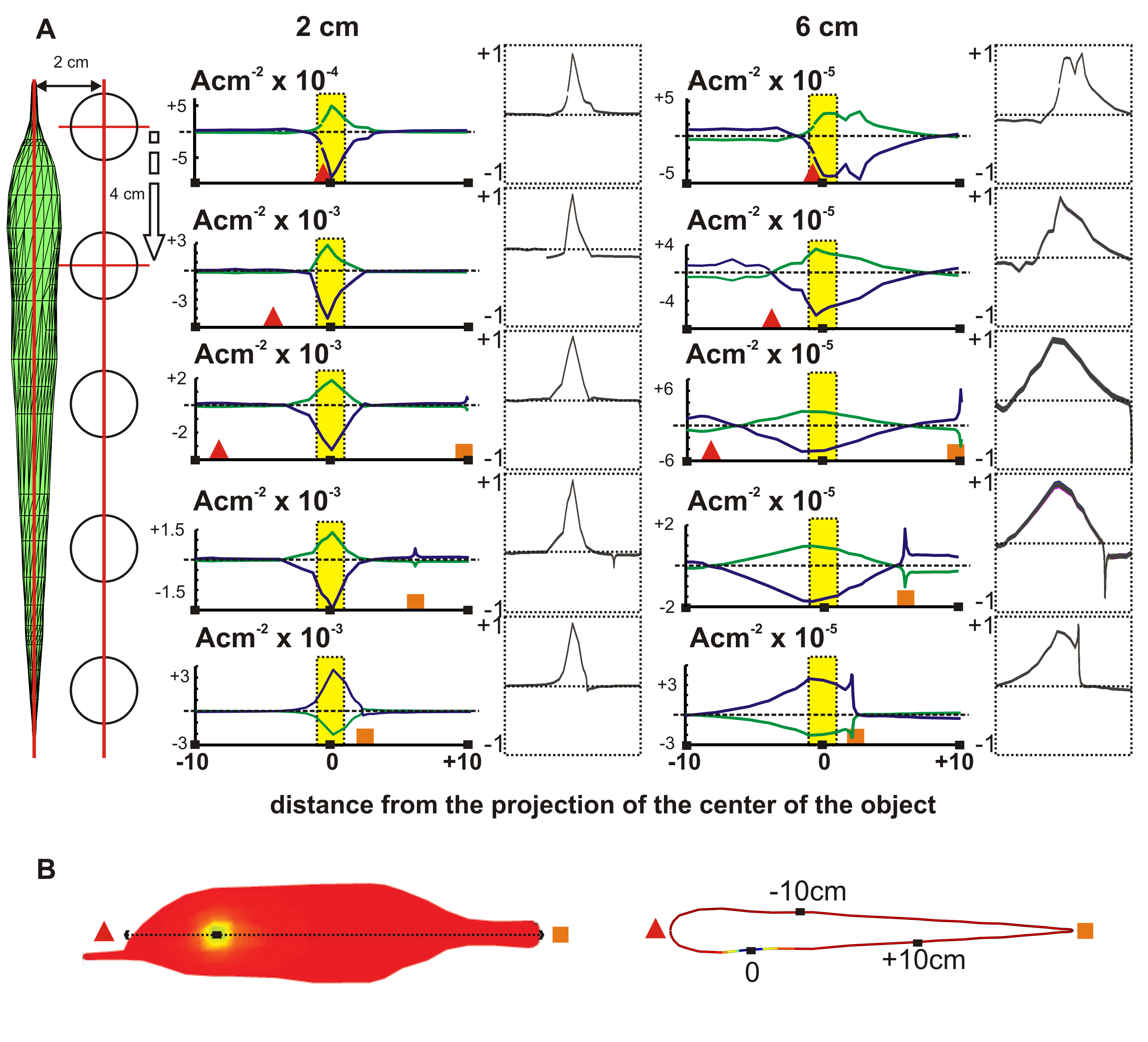

Supplement: Figure S4 — Images of a sphere at 5 points on the side of G. petersii. (A) The diagram shows the relative position of the sphere for each row, when the distance to the longitudinal axis is 2 cm. Each row shows the image profiles for spheres at 2 and 6 cm from the sagittal plane and at different positions along the longitudinal axis shown in the diagram. The plots show the profiles at the peaks of the htEOD waves : positive peak (green) and negative peak (blue). Insets show the superposition of normalized profiles (divided by its maximum absolute value along the EOD). The triangles and squares indicate the fovea and the tail tip respectively in each plot and the yellow area indicates the object projection on the skin. (B) Schematic representation of the localization of the skin section in a lateral view and seen from above, for a sphere placed close to the rostral region. (TIF) [file pcbi.1003722.s004.tif]

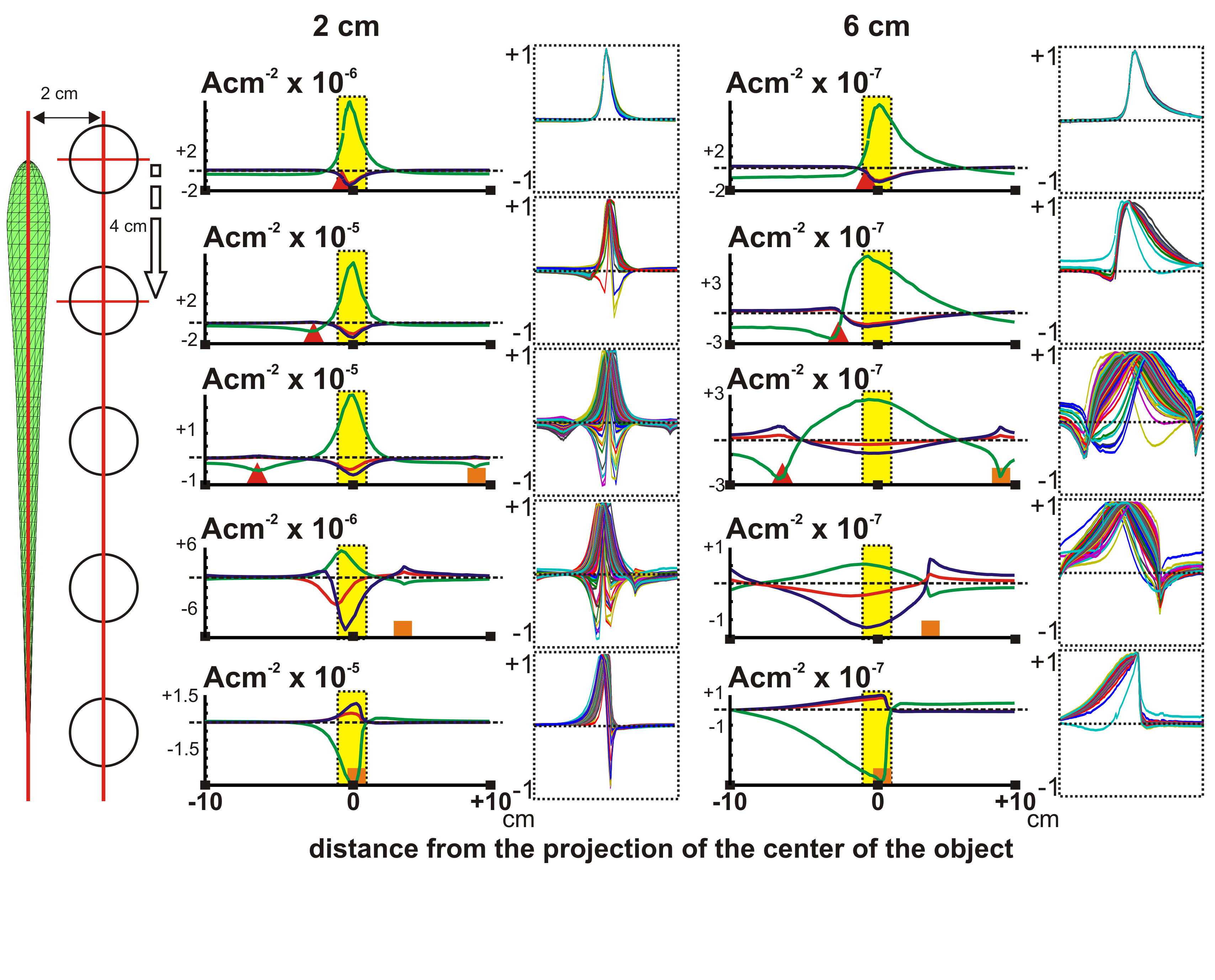

Supplement: Figure S5 — Images of a sphere at 5 points along the side of G. omarorum . Scheme and profiles as in Figure S4. The plots show the profiles at the peaks of the htEOD waves: negative peak (red), positive peak (green) and last negative peak (blue). Insets show the superposition of normalized profiles along the EOD. Note that the shapes of the images at the peaks of the waves differ mainly in the 4th line. (TIF) [file pcbi.1003722.s005.tif]

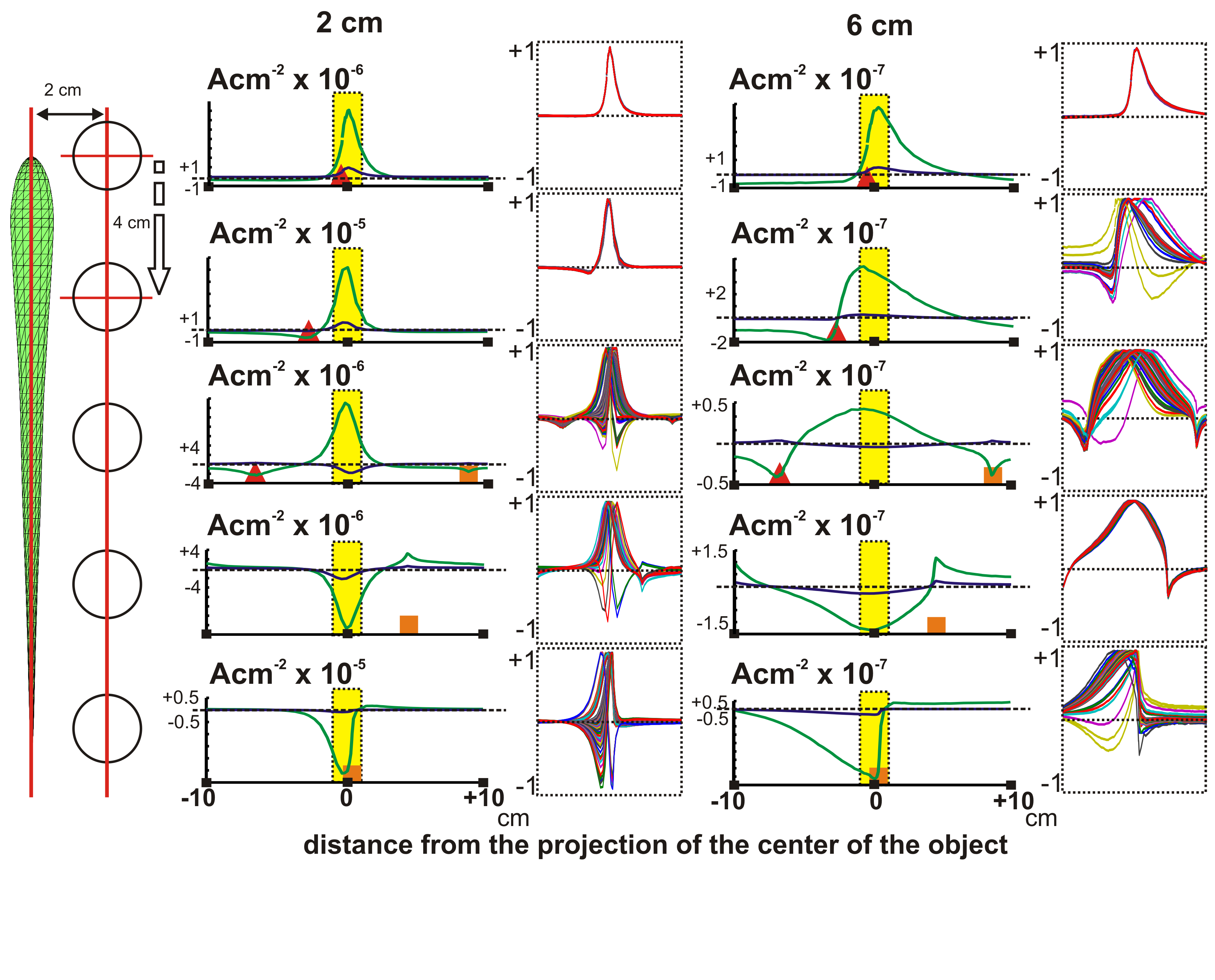

Supplement: Figure S6 — Images of a sphere at 5 points along the side of G. obscurus . Scheme and profiles as in Figure S4. The plots show the profiles at the peaks of the htEOD waves, positive peak (green) and negative peak (blue). Insets show the superposition of normalized profiles along the EOD. Note that the shapes of the images at the peaks of the waves differ mainly in the 3rd line. (TIF) [file pcbi.1003722.s006.tif]

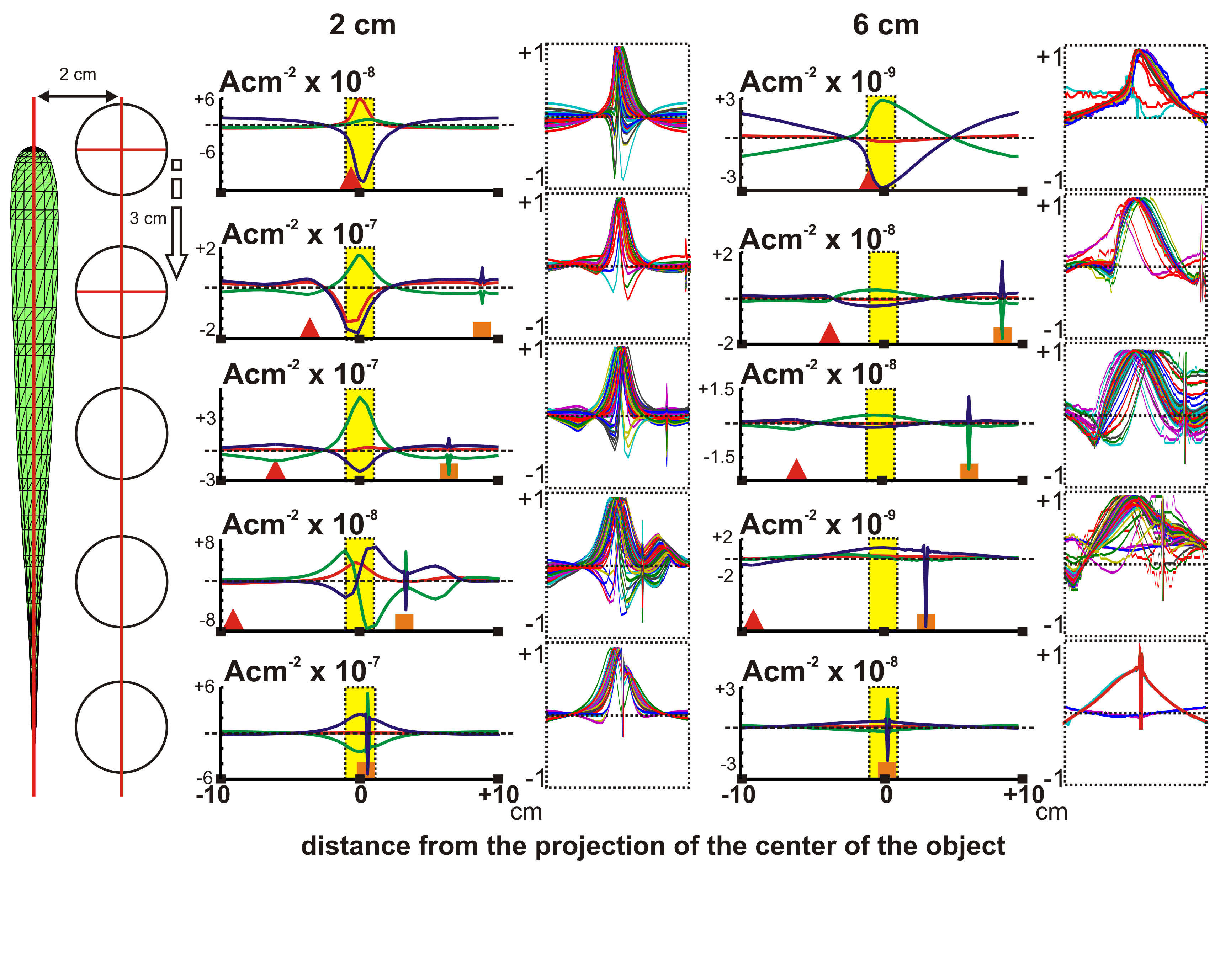

Supplement: Figure S7 — Images of a sphere at 5 points along the side of G. coropinae . Scheme and profiles as in Figure S4.The plots show the profiles at the peaks of the htEOD waves: first negative peak (red), positive peak (green) and last negative peak (blue). Insets show the superposition of normalized profiles along the EOD. Note that the shapes of the images at the peaks of the waves differ mainly in the 4th line. (TIF) [file pcbi.1003722.s007.tif]

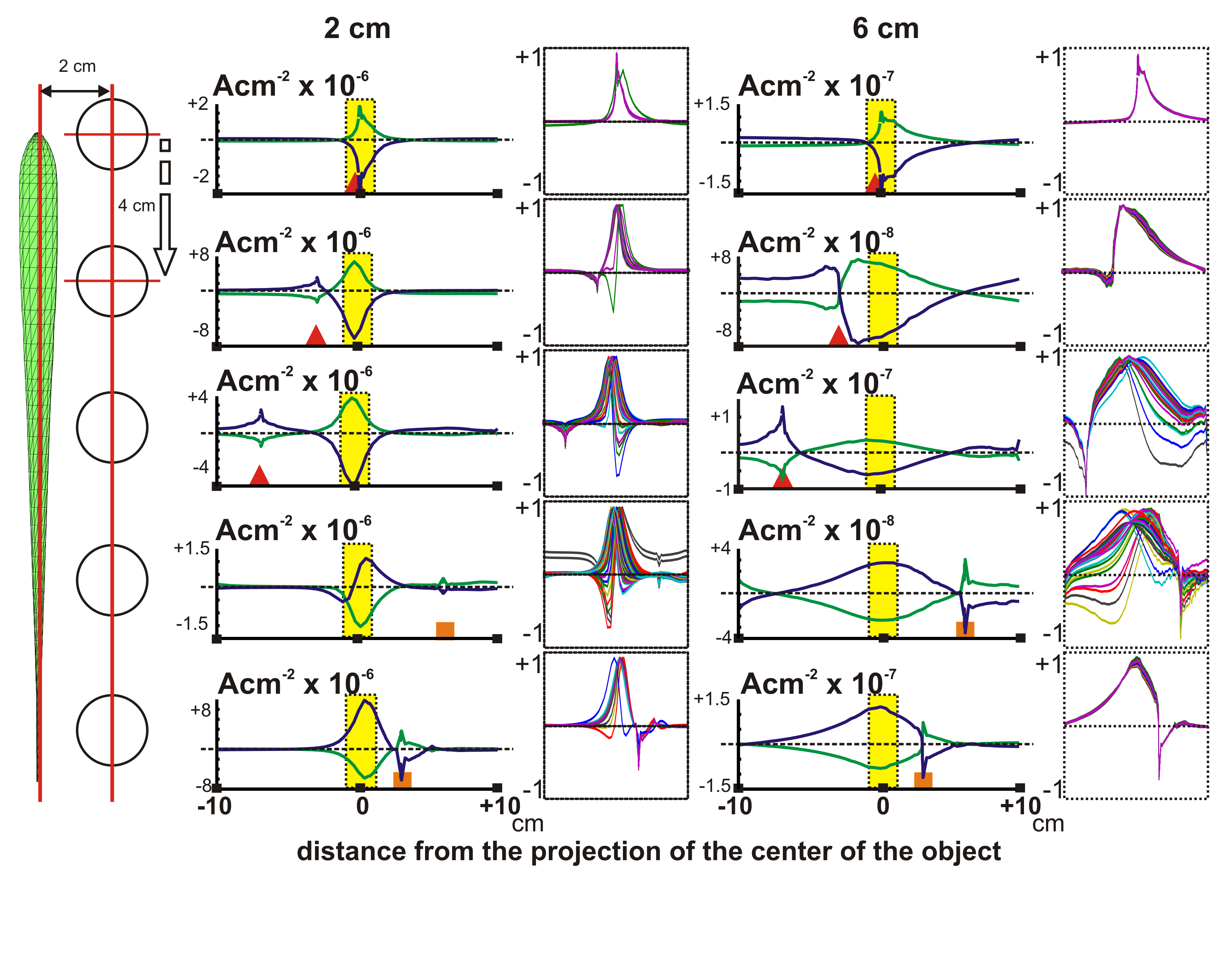

Supplement: Figure S8 — Images of a sphere at 5 points along the side of A. albifrons . Scheme and profiles as in Figure S4.The plots show the profiles at the peaks of the htEOD waves: positive peak (green) and negative peak (blue). Insets show the superposition of normalized profiles along the EOD. Note that the shapes of the images at the peaks of the waves differ mainly in the 4th line. (TIF) [file pcbi.1003722.s008.tif]

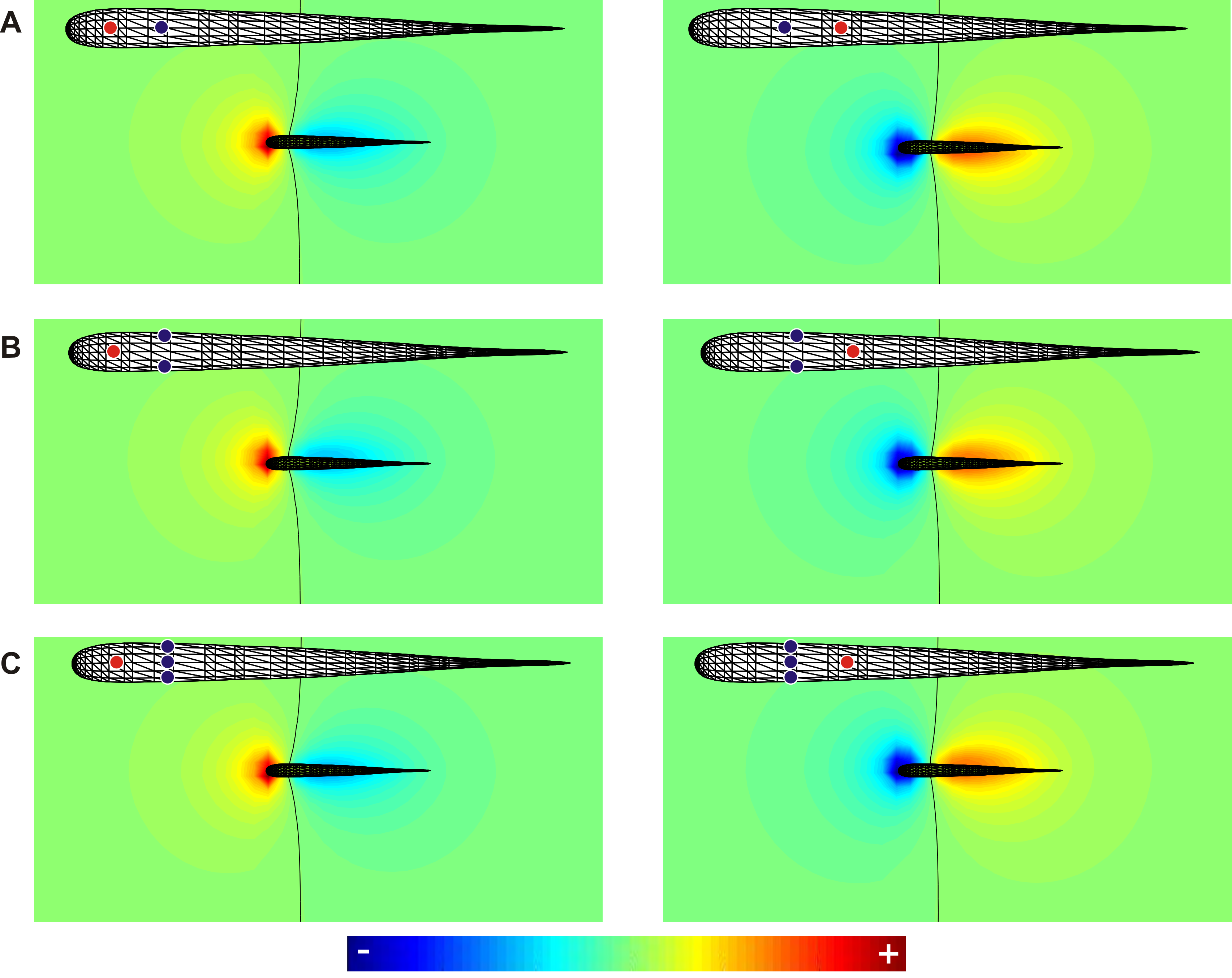

Supplement: Figure S9 — Comparison of different models for EOs in G. coropinae . Color maps show external potentials calculated using a single dipole (A), two symmetrical dipoles (B) and three symmetrical dipoles (C). (TIF) [file pcbi.1003722.s009.tif]
